# Effects of Si and Sr elements on solidification microstructure and thermal conductivity of Al–Si-based alloys

**DOI:** 10.1007/s10853-022-07045-7

**Published:** 2022-03-08

**Authors:** Xun Zhang, Yuli Zhou, Gu Zhong, Junchao Zhang, Yunan Chen, Wanqi Jie, Peter Schumacher, Jiehua Li

**Affiliations:** 1 Institute of Casting Research, Montanuniversität Leoben, 8700 Leoben, Austria; 2 State Key Laboratory of Solidification Processing, Northwestern Polytechnical University, Xi’an, 710072 China; 3 Chinalco Materials Application Research Institute Co., Ltd., Suzhou Branch, Suzhou, 215026 China; 4 Austrian Foundry Research Institute, 8700 Leoben, Austria

## Abstract

Effects of Si and Sr on solidification microstructure and thermal conductivity of Al–Si binary alloys and Al–9Si–Sr ternary were investigated, respectively, with a special focus on the relationship between solidification microstructure and thermal conductivity. It was found that (i) in Al–Si binary alloys, with increasing Si content, α-Al grain size increases and then decreases when Si content is over 7 wt%, while the percentage of eutectic Si continuously increases, which significantly decreases the thermal conductivity and electrical conductivity, and (ii) in Al–9Si–Sr ternary alloys, the presence of Sr has no significant effect on α-Al grain, but effectively modifies eutectic Si and significantly improves the thermal and electrical conductivity. On this basis, two theoretical calculation models [the Maxwell model and the Hashin–Shtrikman (H–S) model] were used to elucidate the relationship between solidification microstructure and thermal conductivity. Compared with the Maxwell model, the H–S model fits better with the measured values. The obtained results are very helpful to the precise composition control during alloy design and recycling of Al–Si-based alloys with the aim to further improve the thermal conductivity of Al–Si-based alloys.

**Graphical abstract:**

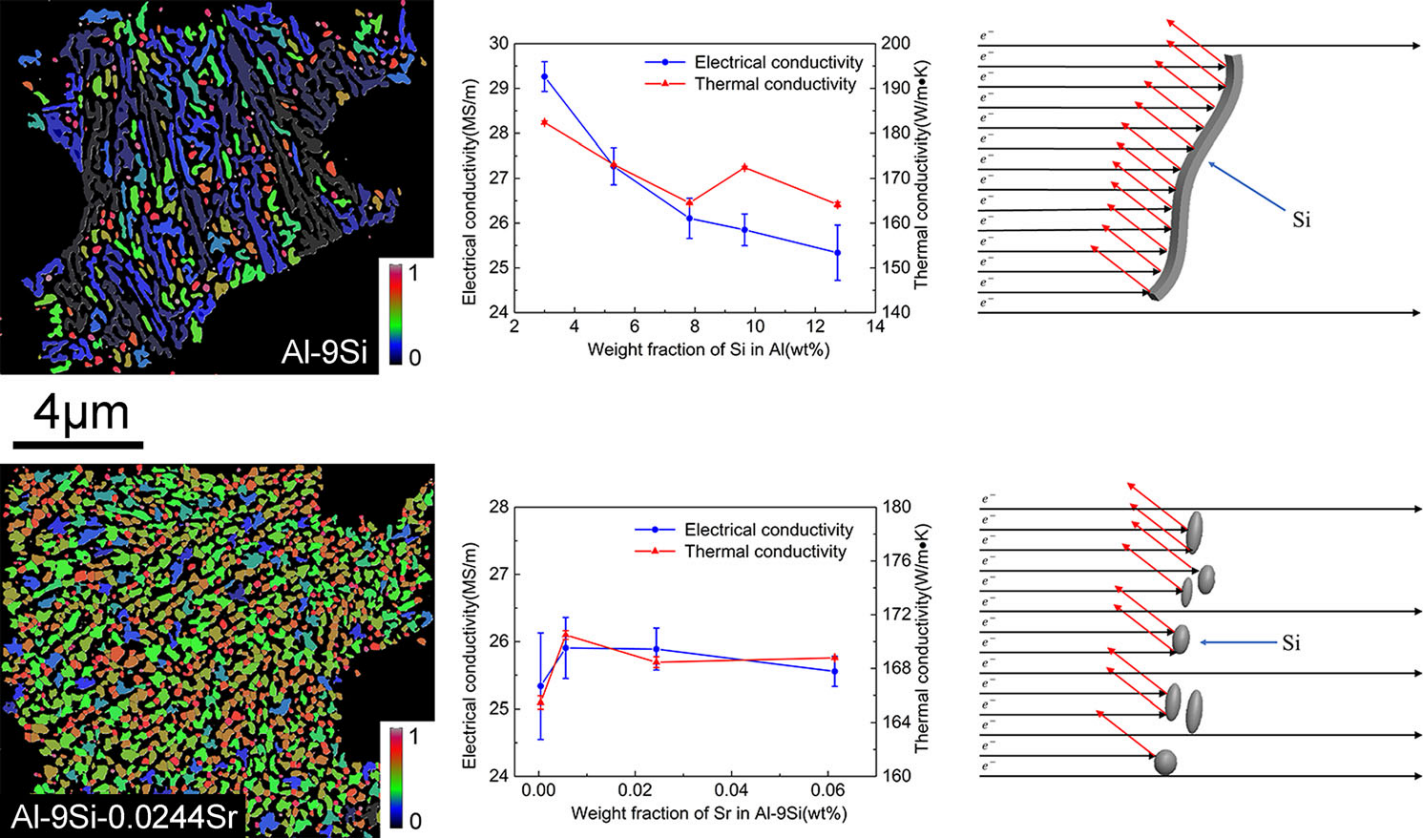

**Supplementary Information:**

The online version contains supplementary material available at 10.1007/s10853-022-07045-7.

## Introduction

Al–Si-based alloy is one of the most important Al-based foundry alloys. Hypoeutectic Al–Si-based alloys commonly contain 4–12 wt% Si and have been widely used due to their excellent casting properties, desirable mechanical properties and low costs [1]. With the improvement of cutting-edge technology in the automotive and communication industries, there is an increasing demand to extend the energy density because of the fact that various casting parts (i.e., LED light equipment and 5G conversation base station heat dissipation gear) require excessive integration, miniaturization and lightweight. Although pure Al has an excellent thermal conductivity (237 Wm^−1^ K), the thermal conductivity of Al–Si-based alloy is generally low. For example, the thermal conductivity of A380 alloy is only 96 Wm^−1^ K, less than half of pure Al, which is inadequate to meet the increasing application demands. It is, therefore, of great necessity to further improve the thermal conductivity of Al–Si-based alloy, which can be directly related to the solidification microstructure.

In terms of solidification microstructure of hypoeutectic Al–Si alloy, it is mainly composed of primary α-Al and eutectic Si. In order to refine the primary α-Al grain, grain refiners such as Al–5Ti–1B and Al–Nb–B have been very often used [2], [2], [2], while in order to modify the eutectic Si, Sr has been very often used [5], [5]. In terms of the thermal and electrical conductivity of hypoeutectic Al–Si alloy, elucidating effects of alloying elements on the thermal and electrical conductivity is of great importance. It has been reported by Chen et al. that the thermal conductivity of Al–Si alloys decreases with increasing Si content up to 6 wt%. When the Si content exceeds 6 wt%, the thermal conductivity basically remains stable at about 157 Wm^−1^ K [7]. It has been reported by Kim et al. that the introduction of Si, Fe, Mg, Mn and Cu decreases the thermal conductivity. Furthermore, Mn element was found to cause the most considerable reduction of the thermal conductivity [8]. Apart from the effect of decreasing Mn content, it has been reported by Lumley et al. [9] that the thermal conductivity of Al–Si–Cu alloys increases by about 60% after T7 heat treatment. It has also been reported by Stadler et al. [10] that the introduction of Cu element leads to the formation of precipitation phases such as θ-Al_2_Cu and Q-Al_5_Cu_2_Mg_8_Si_6_ at 250 °C, which decreases the thermal conductivity of Al alloys. The effects of other elements on the thermal conductivity of Al alloys were also discussed [5], [5]. Apart from effects of alloying elements, the thermal conductivity of Al–Si-based alloys is also influenced by the morphology (shape and size) of eutectic Si which is very often achieved by the modification of Sr or solution treatment. It has been reported by Mulazimolu et al. that the Sr element itself does not affect the thermal conductivity of Al alloys, but the modification of eutectic Si caused by Sr does significantly increase the thermal conductivity of Al alloys. The thermal conductivity difference between Al–Si–Sr alloys and Al–Si alloys increases continuously with increasing Si content, reaching 11.8 pct IACS at the point of eutectic composition (about 12 wt% Si) [12].

Clearly, Si is the one of the most important alloying elements in Al–Si alloy, and its content, size, shape and distribution have an important influence on the thermal conductivity of Al–Si-based alloy. It should be noted that most previous researches use commercial alloys with different impurity levels such as V, Mn, Cr, Ti and other transition group elements, which can seriously affect the thermal conductivity. More importantly, the presence of such impurities also makes it difficult to elucidate effects of Si and Sr elements on the thermal conductivity of Al–Si-based alloys.

In this paper, high-purity Al–Si-based alloys were used in order to elucidate effects of different Si contents in Al–Si binary alloys and different Sr contents in Al–9Si–Sr ternary alloys on the solidification microstructure and thermal conductivity of Al–Si-based alloy, with a special focus on the relationship between solidification microstructure and thermal conductivity. This investigation is aimed to provide theoretical basis for further improving the thermal conductivity of Al–Si-based alloys.

## Experiments and methods

### Casting sample preparation

Al–Si binary alloys with different Si contents (3 wt% Si, 5 wt% Si, 7 wt% Si, 9 wt% Si, 12 wt% Si, wt%, used through the paper unless noted) and Al–9 wt% Si–Sr ternary alloys (with different Sr levels of 4 ppm, 56 ppm, 244 ppm, 614 ppm) were prepared by melting high-purity Al (99.97%), high-purity Si (99.998%), and Al–10Sr master alloy in a resistance furnace at 800 °C. The melt was stirred with a ceramic rod and kept at 800 °C for at least 30 min. After removing the dross from the surface, the melt was poured into a steel mold to produce a cast bar of 15 (width) × 50 (length) × 140 mm (height). The chemical composition of the samples was measured by OBLF VeOS photoemission spectrometry, as listed in Table 1. The cooling curves were recorded by using a *K*-type thermocouple that was positioned in the middle of a QC4080-QK500 mold with a height of about 40 mm, a width of 35 mm, and a length of 35 mm. The temperature statistics have been recorded in a Heraeus Electro-Nite thermal analysis unit and examined by the PicoLog recorder software.

**Table 1:** Measured compositions of the alloys

Sample no.	Si (wt%)	V (ppm)	Sr (ppm)	Mn (ppm)	Cr (ppm)	Al
Al–3Si	3.000	< 10	< 1	< 2	< 10	Bal
Al–5Si	5.299	< 10	< 1	< 2	< 10	Bal
Al–7Si	7.824	< 10	< 1	< 2	< 10	Bal
Al–9Si	9.650	< 10	< 1	< 2	< 10	Bal
Al–12Si	12.74	< 10	< 1	< 2	< 10	Bal
Al–9Si–0.0004Sr	9.273	< 10	4	< 2	< 10	Bal
Al–9Si–0.0056Sr	9.347	< 10	56	< 2	< 10	Bal
Al–9Si–0.0244Sr	9.606	< 10	244	< 2	< 10	Bal
Al–9Si–0.0614Sr	9.286	< 10	614	< 2	< 10	Bal

### Microstructure analysis

**Figure 1:**
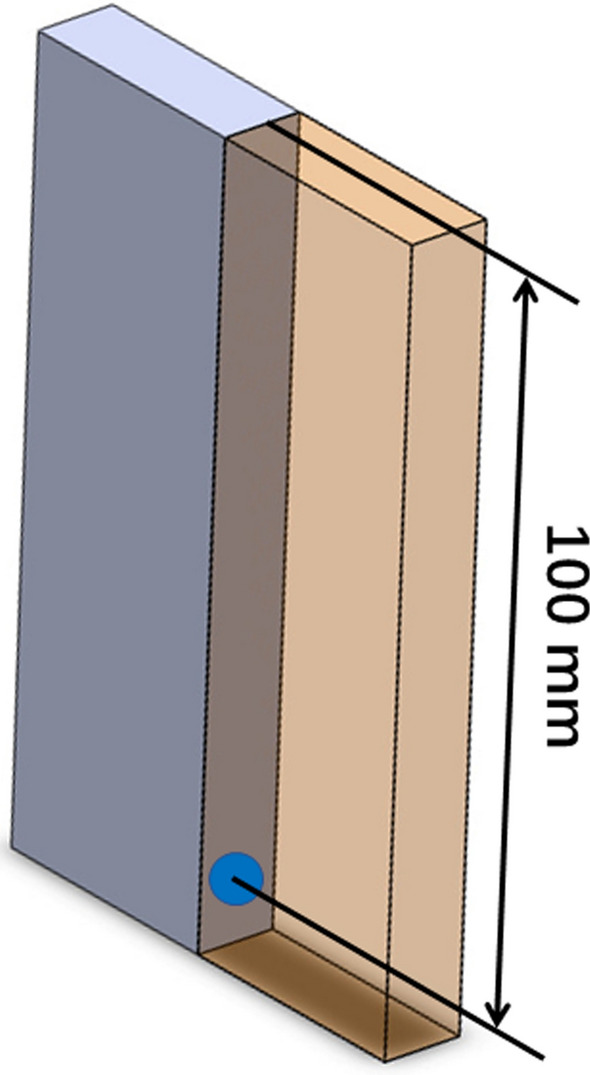
Schematic diagram of sampling locations

The sample was cut from the center, and the area nearby the bottom of the cut surface, as shown in the blue area of Fig. 1, was taken for solidification microstructure characterization. The samples were prepared by successively polishing with 80#, 320#, 800# and 1200# sandpaper, then polishing with 3-μm diamond polishing solution for half an hour and finally polishing with 50-nm SiO_2_ polishing solution. The prepared metallographic samples were etched with Barker’s reagent (13 g boric acid + 35 g HF + 800 ml H_2_O), and the etching time is about 2 min. The metallographic microstructure of the samples after etching was observed with a Zeiss AXIO optical microscope. The average size of α-Al grains was measured using the linear intercept method. It should be noted that a total of 16 (4 × 4) individual photographs were synthesized into one photograph by the photo acquisition software in order to obtain statistical grain sizes within a larger area.

After the metallographic analysis, the sample was put into the special holder with a fixed size for electron probe microanalyzer (EPMA), and then, the etching layer on the surface of the sample was firstly removed with 1200# sandpaper, then polished with diamond grinding solution of 3 μm particle size for half an hour, and finally polished with SiO_2_ polishing solution of 50 nm particle size. The solubility of each element within the α-Al matrix and eutectic region was measured by EPMA (JEOL-JXA-8200F).

The eutectic Si morphology was observed using SEM (FEI-Quanta 200). For the volume fraction of the eutectic Si, SEM images with 1000× magnification were used, while for the particle size and shape factor SEM images with 6000× magnification were used. All SEM images were quantified by ImageJ software.

Furthermore, the morphology of Si particle can be characterized by shape factor F, which can be expressed as (Eq. 1):

1 $$F = \frac{4\pi \times S}{{L^{2} }}$$

where *S* is the area of a connected region and *L* is its perimeter [13]. The value of *F* is between 0 and 1. The closer the value is to 1, the closer the particle shape is to be spherical.

### Electrical and thermal conductivity

As shown in Fig. 1, the samples for measuring thermal conductivity were taken from the middle of the microstructure characterization part. The samples were accurately machined to 10 mm (length) × 10 mm (width) × 3 mm (height), and then, the surface was polished. The thermal conductivity of the samples was measured by the flash laser method according to Eq. 2 [14]:

2 $$\lambda = \alpha \times \rho \times C_{p}$$

where *λ* is the thermal conductivity (Wm^−1^ K), α is the thermal diffusivity (mm^2^ s^−1^), *ρ* is the density (g/cm^−3^) and *C*_*p*_ is the specific heat of the sample at room temperature (Jg^−1^ K). It has been reported that the specific heat measured by differential scanning calorimetry and the density measured by density balance instrument of Al–Si-based alloy at room temperature are close to that of pure Al, about 0.88 Jg^−1^ K and 2.7 g/cm^−3^, respectively [8]. The thermal diffusivity was measured using a Netzsch LFA 467 laser flash meter in accordance with ASTM E1461. Each sample was tested at least 3×. The reported value is the average value with its standard deviation. In order to further verify the results of thermal conductivity, the electrical conductivity of the sample was also measured by SIGMATEST eddy current conductivity meter at room temperature. Each sample was also tested at least three times. The reported value is the average value with its standard deviation.

## Results

### Thermal analysis

**Figure 2:**
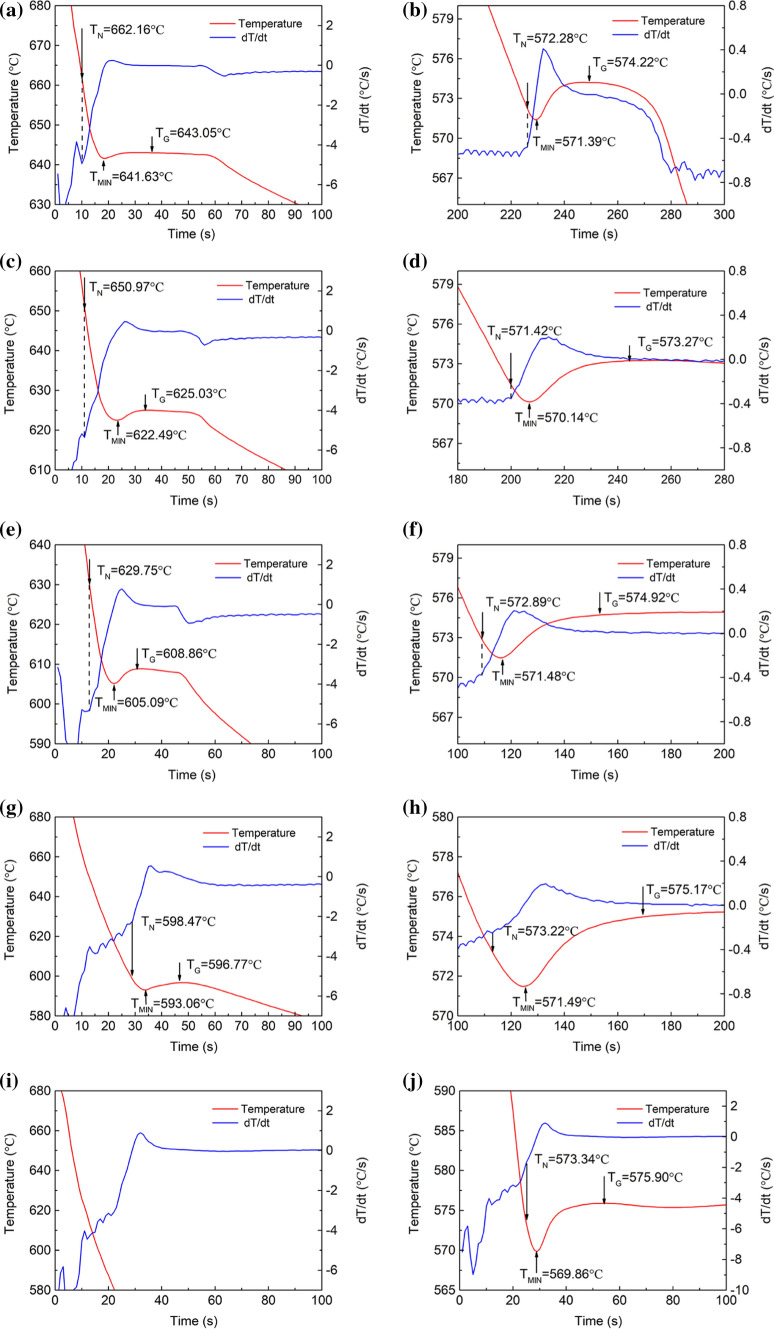
Characteristic temperatures of Al–Si binary alloys. (**a**, **b**) Al–3Si, (**c**, **d**) Al–5Si, (**e**, **f**) Al–7Si, (**g**, **h**) Al–9Si, (**i**, **j**) Al–12Si. (**a**, **c**, **e**, **g**) are for α-Al, while (**b**, **d**, **f**, **h**) are for eutectic Si

**Figure 3:**
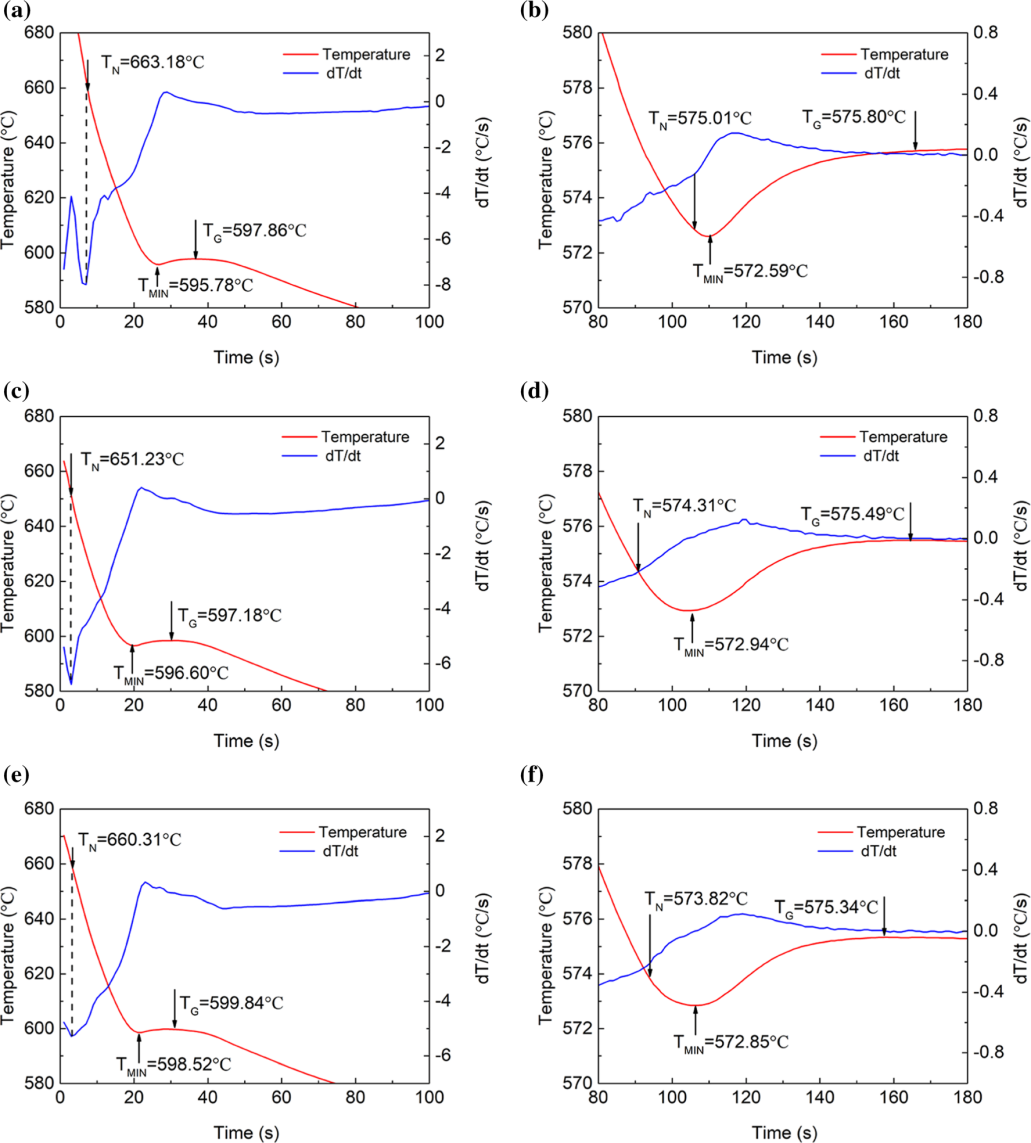
Characteristic temperatures of Al–9Si–Sr ternary alloys with the additions of (**a**, **b**) 4 ppm Sr, (**c**, **d**) 244 ppm Sr, (**e**, **f**) 614 ppm Sr. (**a**, **c**, **e**) are for α-Al, while (**b**, **d**, **f**) are for eutectic Si

The solidification process is often accompanied by the release or absorption of heat and is reflected in the cooling curve of the sample. As shown in Figs. 2 and 3, the values of nucleation temperature (*T*_N_), minimum temperature (*T*_min_), growth temperature (*T*_G_) and latent heat of solidification can be determined from the cooling curve and temperature–time first-order differential curve, respectively. The temperature where the slope of the differential curve begins to increase abruptly is defined as the nucleation temperature (*T*_N_). When nucleation occurs, the latent heat of solidification releases, and the trend of temperature reduction slows down. As the nucleation rate increases, the latent heat of crystallization gradually increases. When the released latent heat of solidification is equal to the amount of heat dissipated by the sample, the rate of temperature reduction is close to zero. This temperature is defined as the minimum temperature (*T*_min_). As the solidification process continues to occur, the latent heat of solidification of the sample continues to be released and the temperature increases. When the latent heat of solidification is balanced with the heat dissipated in the cooling process, the temperature increase rate is close to zero, and the growth temperature (*T*_G_) is reached. It should be noted that the equilibrium nucleation temperature is calculated by Thermo-Calc software with TCAL5 database. The difference between the calculated nucleation temperature and the measured nucleation temperature is defined as the nucleation undercooling (Δ*T*). The obtained results in Al–Si binary alloys and Al–9Si–Sr ternary alloys are listed in Table 2 and Table 3, respectively.

**Table 2:** Characteristic temperatures of Al–Si binary alloys (°C)

Designation		*T*_N_/°C	*T*_Min_/°C	*T*_G_/°C	Δ*T*	Δ*T*_RU_/°C
α-Al	Al–3Si	662.16	641.63	643.05	0.92	1.42
	Al–5Si	650.97	622.49	625.03	6.30	2.54
	Al–7Si	629.75	605.09	608.86	10.76	3.77
	Al–9Si	598.47	593.06	596.77	8.90	3.71
	Al–12Si	–	–	–	–	–
Eutectic Si	Al–3Si	572.28	571.39	574.22	4.95	2.83
	Al–5Si	571.42	570.14	573.27	5.86	3.13
	Al–7Si	572.89	571.48	574.92	4.53	3.44
	Al–9Si	573.22	571.49	575.17	3.78	3.68
	Al–12Si	573.34	569.86	575.90	5.21	6.04

**Table 3:** Characteristic temperatures of Al–9Si–Sr ternary alloys (°C)

Designation		*T*_N_/°C	*T*_Min_/°C	*T*_G_/°C	Δ*T*	Δ*T*_RU_/°C
α-Al	Al–9Si–0.0004Sr	663.18	595.78	597.86	6.43	2.08
	Al–9Si–0.0244Sr	651.23	596.60	598.47	5.82	1.87
	Al–9Si–0.0614Sr	660.31	598.52	599.84	4.11	1.32
Eutectic Si	Al–9Si–0.0004Sr	575.01	572.59	575.80	3.81	3.21
	Al–9Si–0.0244Sr	574.31	572.94	575.49	3.28	2.55
	Al–9Si–0.0614Sr	573.82	572.85	575.34	3.68	2.49

In Al–Si binary alloys, in terms of α-Al, with increasing Si content from 3 to 9wt%, *T*_N_ decreases continuously from 662.16 to 598.47 °C, Δ*T* increases from 0.92 to 8.9 °C, and the recalescence temperature increases from 1.42 to 3.71 °C. In terms of eutectic Si, with increasing Si content, no significant change of *T*_N_ and Δ*T* was observed, but the recalescence temperature increases from 2.83 to 6.04 °C.

In Al–9Si–Sr ternary alloys, in terms of α-Al, with increasing Sr content, no significant change of *T*_N_ was observed, but the recalescence temperature decreases from 2.08 to 1.32 °C. In terms of eutectic Si, with increasing Sr content, the nucleation temperature decreases from 575.01 °C with 4 ppm Sr to 574.82 °C with 614 ppm Sr, and the recalescence temperature decreases from 3.21 to 2.49 °C, respectively.

### Solidification microstructure

#### Grain size of α-Al

**Figure 4:**
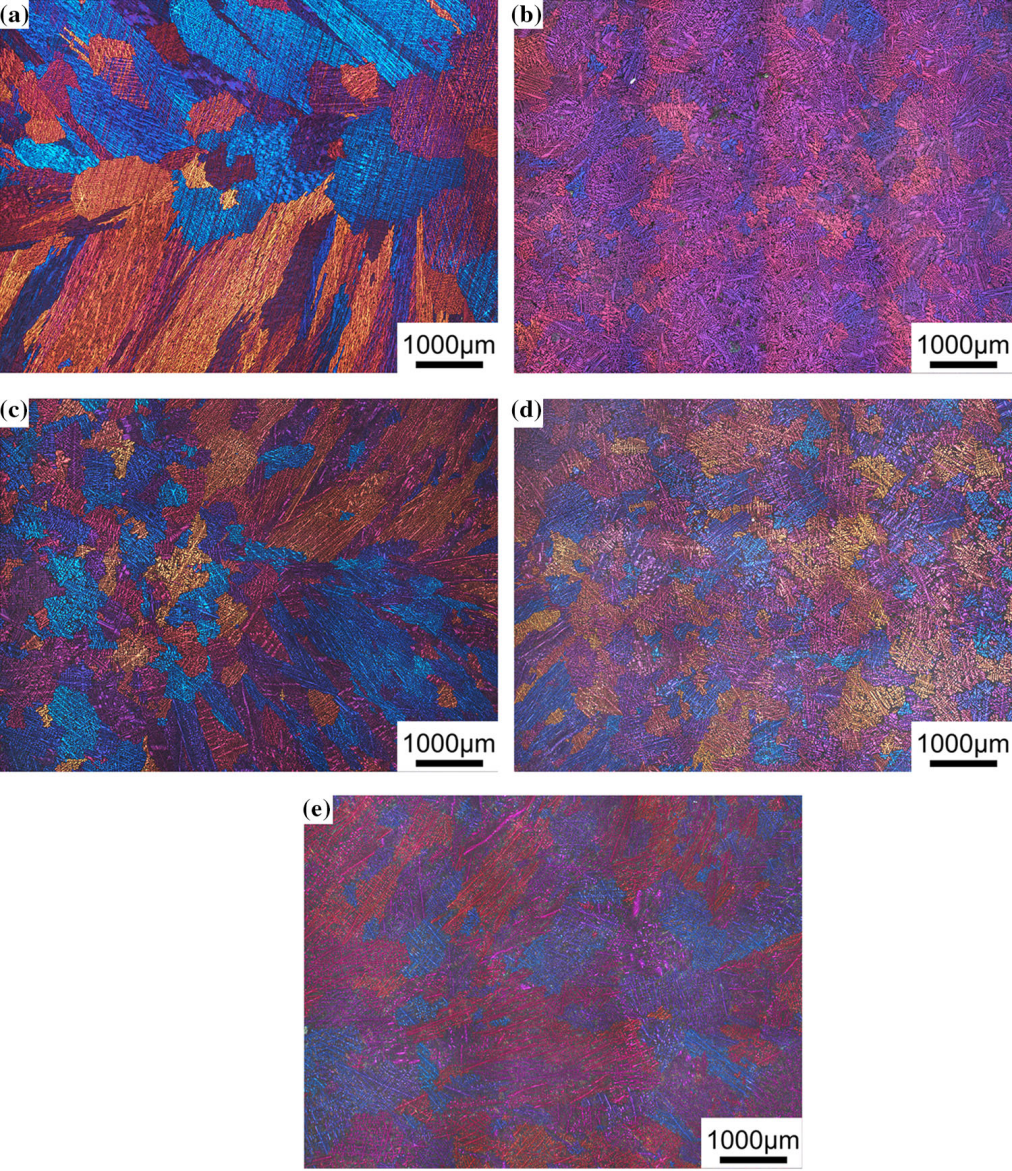
Typical metallographic optical microscopy images of Al–Si binary alloys. **a** Al–3Si, **b** Al–5Si, **c** Al–7Si, **d** Al–9Si, **e** Al–12Si

**Figure 5:**
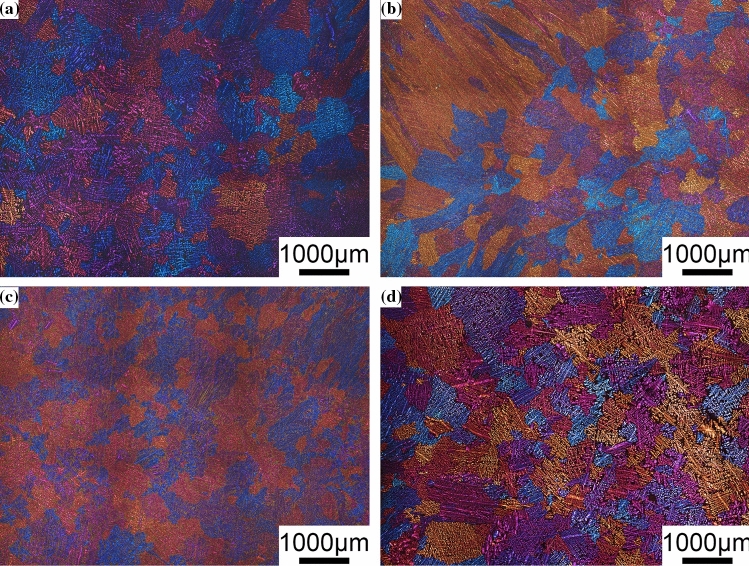
Typical metallographic optical microscopy images of Al–9Si–Sr ternary alloys with the addition of **a** 4 ppm Sr, **b** 56 ppm Sr, **c** 244 ppm Sr, **d** 614 ppm Sr

**Figure 6:**
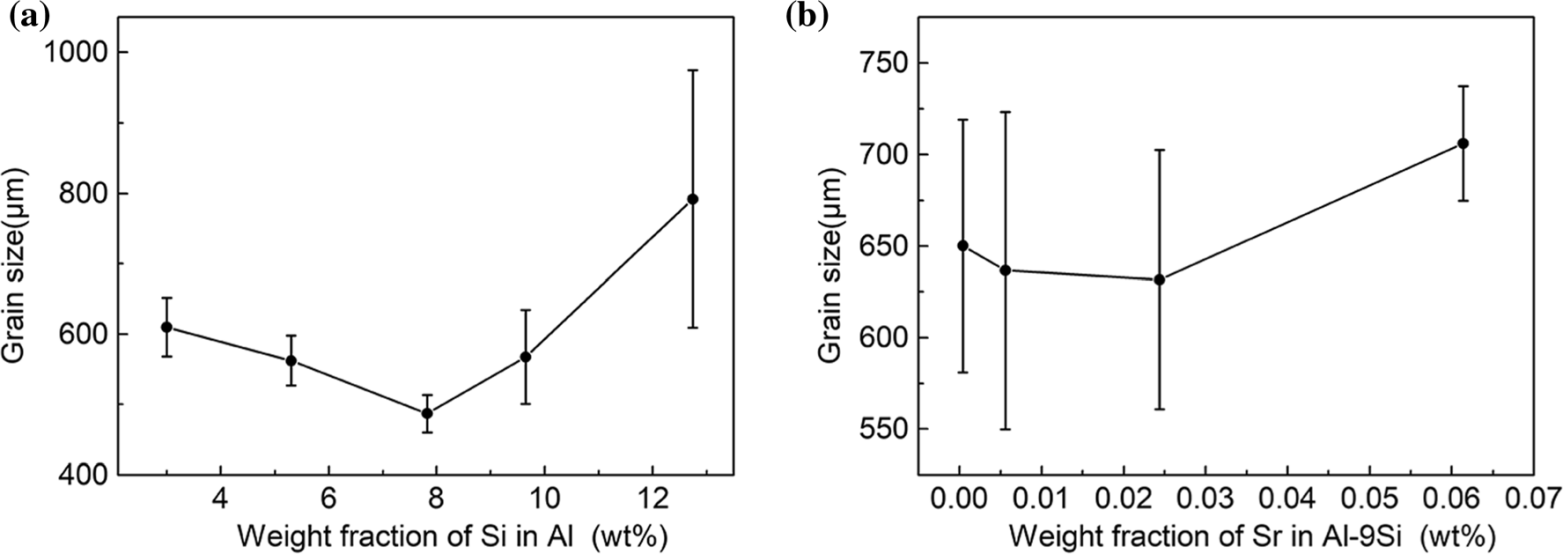
The measured grain size of **a** Al–Si binary alloys and **b** Al–9Si–Sr ternary alloys

Figures 4 and 5 show typical metallographic optical microscopy images of the Al–Si binary alloys and Al–9Si–Sr ternary alloys, respectively. Figure 6 shows the measured α-Al grain size. In Al–Si binary alloys, the α-Al grain size decreases and then increases with increasing Si content from 3 to 12 wt%. The minimum value of 487 μm is obtained when the Si content is 7 wt%. In Al–9Si–Sr ternary alloys, the grain size increases from 650 to 706 μm with increasing Sr content from 4 to 614 ppm. Taking the statistical error into consideration, it can be concluded that the addition of Sr element has no significant effect on the α-Al grain size of Al–9Si–Sr ternary alloy.

#### Determined contents of Si and/or Sr within α-Al and eutectic area

**Figure 7:**
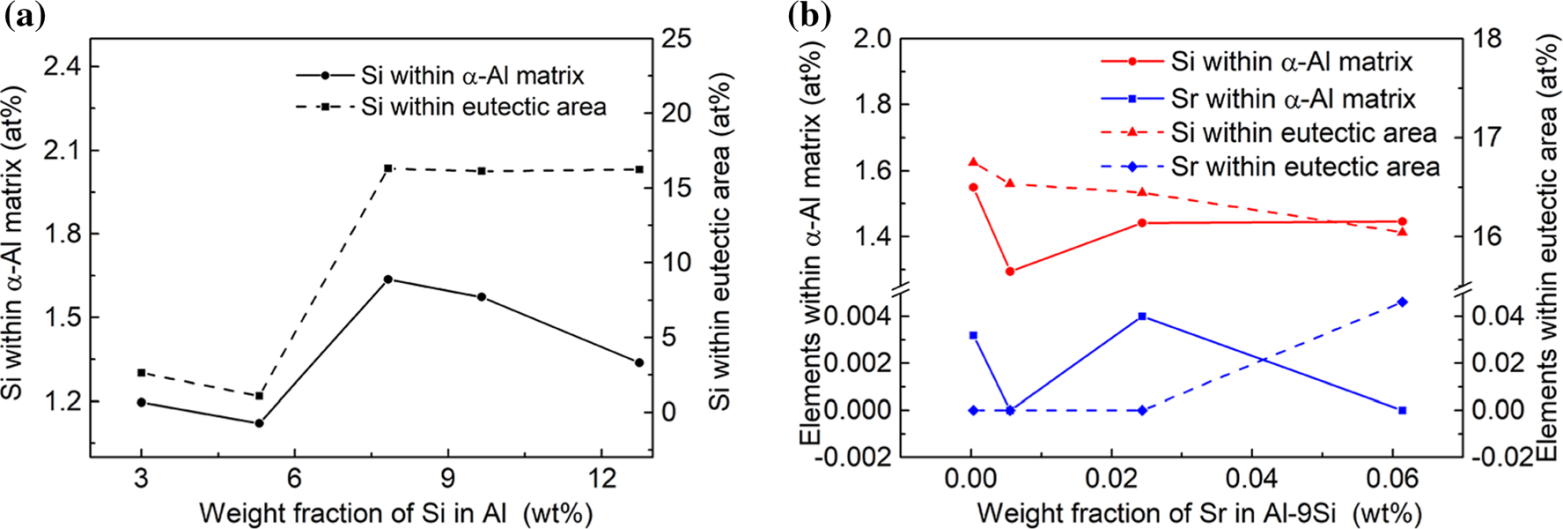
Detected concentrations of Si and Sr in α-Al matrix and eutectic area in **a** Al–Si binary alloys and **b** Al–9Si–Sr ternary alloys

Since the spatial resolution of EPMA for compositional analysis (the smallest area for microdomain compositional analysis) is in the range of a few microns, the determined contents of Si and Sr within the α-Al matrix and eutectic areas can be determined directly by EPMA. To ensure the comparability of measured results, Al matrix and eutectic areas with similar dimensions in all alloys were selected, as shown in Figs. S1 and S2. At least three datasets were measured for each sample. Figure 7 shows the measured average determined contents of elements within the α-Al matrix and eutectic area. In the Al–Si binary alloys, with increasing Si content from 3 to 12wt%, the determined contents of Si in both α-Al matrix and eutectic area increase. The determined content increases from 1.1961 to 1.3368 at.% in α-Al matrix and from 2.6211 to 16.2452 at.% in eutectic area. In the Al–9Si–Sr ternary alloys, with increasing Sr content from 4 to 614 ppm, no significant change of the determined content of Sr element within α-Al matrix was observed, while the determined content of Sr element within the eutectic area increases from 0 to 0.0462 at.%.

#### Microstructure of eutectic Si

**Figure 8:**
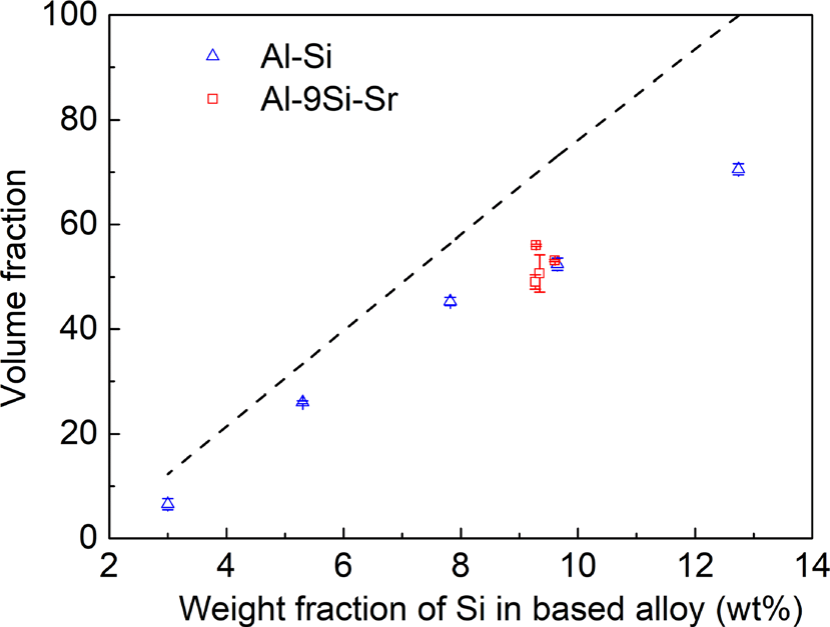
Volume fraction of eutectic Si in Al–Si binary alloys and Al–9Si–Sr ternary alloys (the dashed line is theoretical values calculated from phase diagrams)

Apart from the α-Al matrix, a large amount of eutectic Si was also observed in Al–Si-based alloys. Si is a semiconductor with a resistivity of 3 × 10^11^ μΩ cm, which is much higher than that of the α-Al matrix. Therefore, the electrical conductivity of Al–Si-based alloys depends not only on the α-Al matrix, but also on the number, distribution, size and morphology of eutectic Si in the alloy. It is therefore of great necessity to carry out quantitative analysis of eutectic Si phase. In order to improve the statistical accuracy, two SEM photographs with a magnification of 1000× under the backscattering (BSE) mode were selected for each alloy, and the results were averaged. Figures S3 and S4 show the SEM photographs of Al–Si binary alloy and Al–9Si–Sr alloy, respectively. The dark area is related to the α-Al matrix, while the white area is related to the eutectic Si, and the dark and white mixed area is related to the eutectic area. Using ImageJ software, the area fraction of the eutectic area and eutectic Si in each sample can be counted according to the different grayscales of each phase, and then, the volume fraction can be obtained using the quantitative metallographic method, as shown in Fig. 8. It can be seen that, in the Al–Si binary alloy, the percentage of the eutectic area gradually increases with increasing Si content, while, in the Al–9Si–Sr ternary alloy, no significant change of the percentage of eutectic area was observed with increasing Sr content. It should be noted that the measured volume fraction of eutectic Si is always slightly less than the calculated value because of the fact that the solidification process is under non-equilibrium conditions.

**Figure 9:**
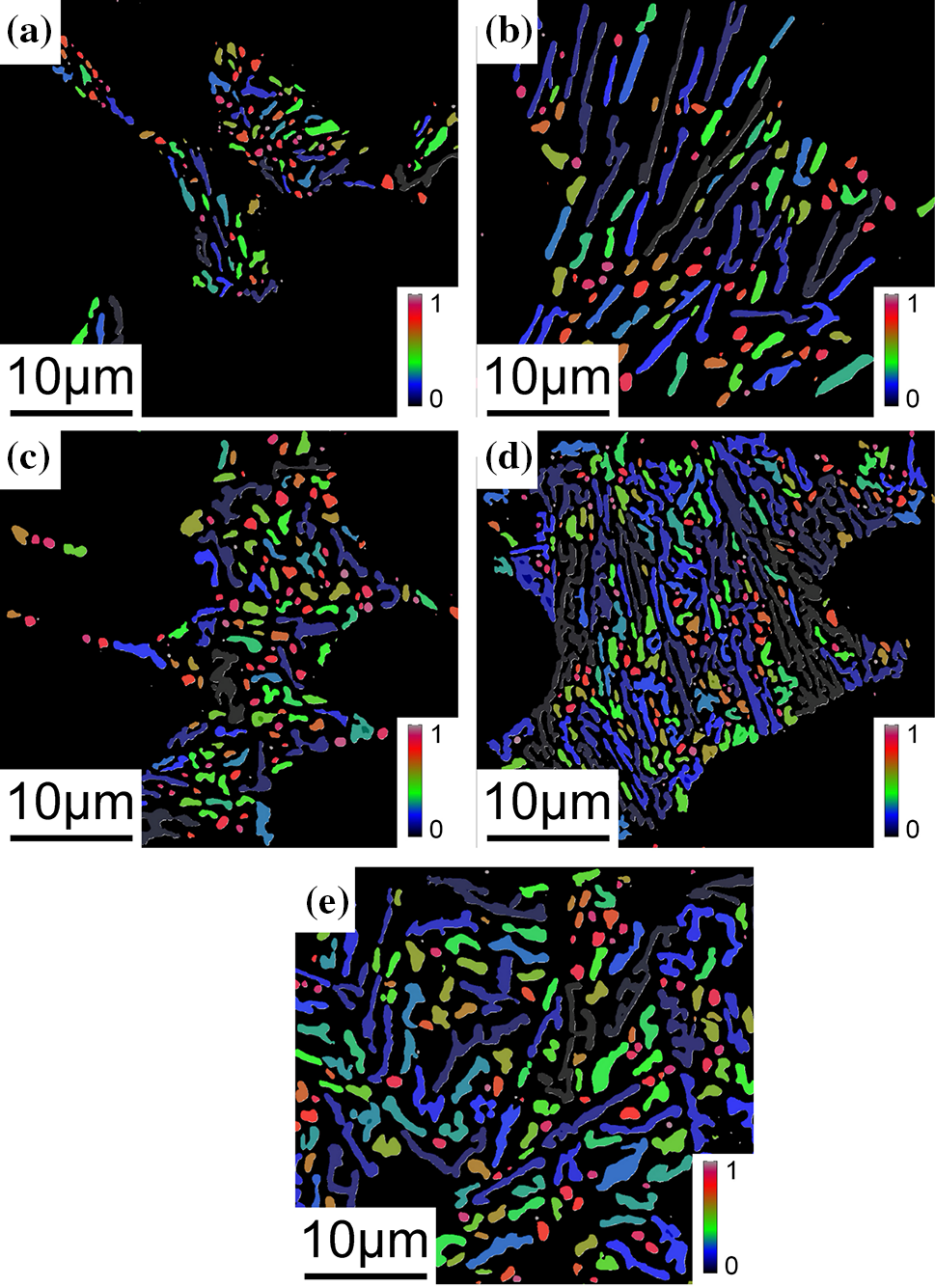
Shape factor of Al–Si binary alloys at 6000 × magnification. (**a**) Al–3Si, (**b**) Al–5Si, (**c**) Al–7Si, (**d**) Al–9Si, (**e**) Al–12Si

**Figure 10:**
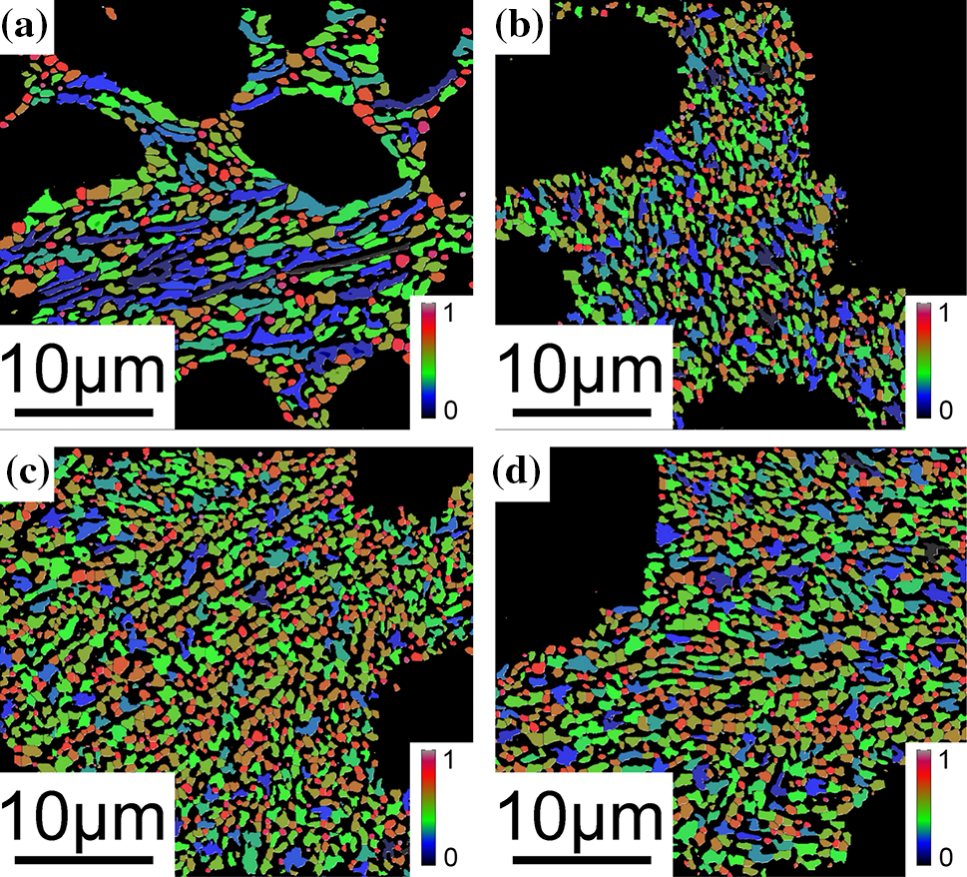
Shape factor of Al–9Si–Sr ternary alloys at 6000 × magnification with the addition of **a** 4 ppm Sr, **b** 56 ppm Sr, **c** 244 ppm Sr, **d** 614 ppm Sr

**Figure 11:**
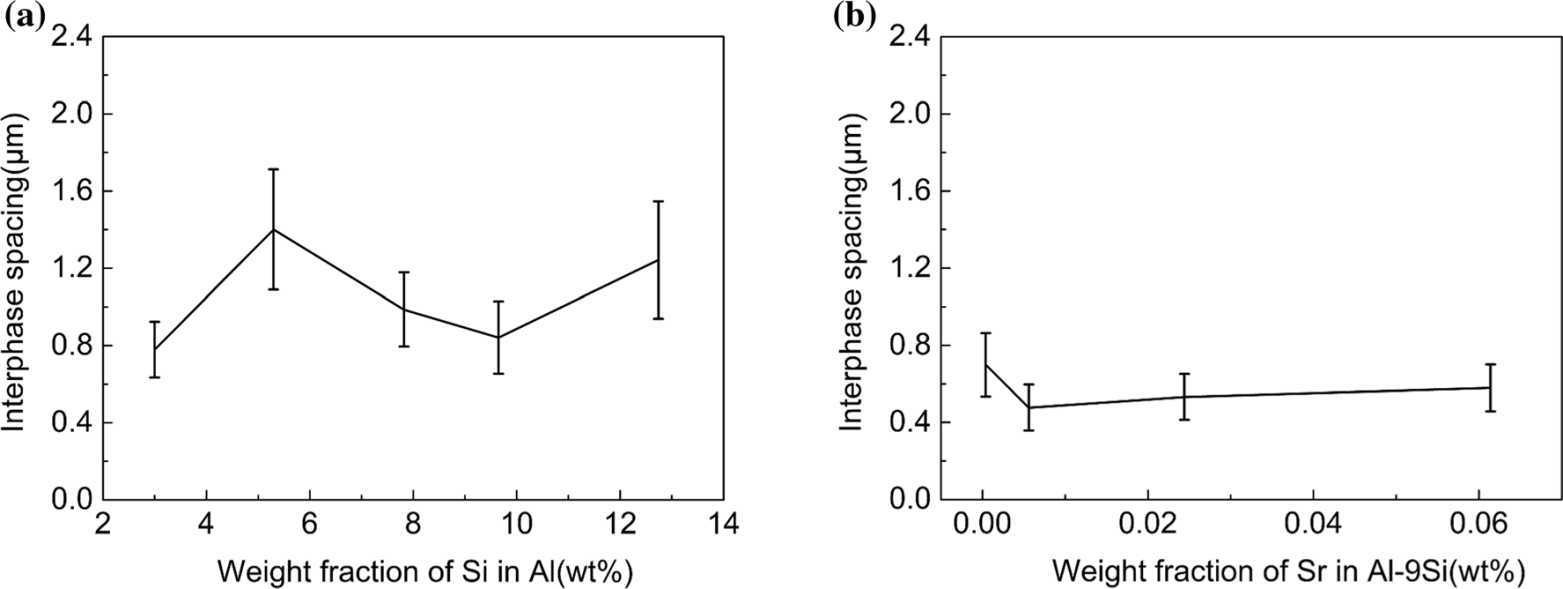
Phase spacing of eutectic Si particles in **a** Al–Si binary alloys and **b** Al–9Si–Sr ternary alloys

**Figure 12:**
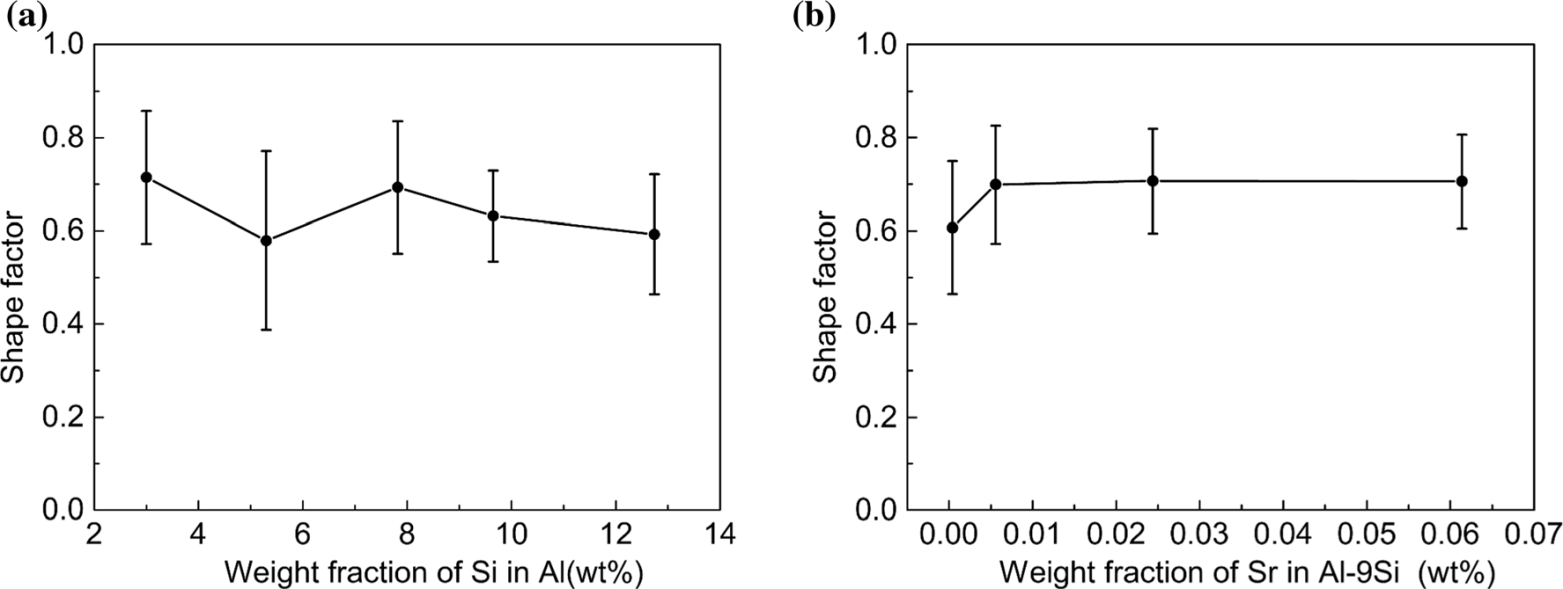
Shape factor of eutectic Si particles in **a** Al–Si binary alloys and **b** Al–9Si–Sr ternary alloys

The SEM magnification was enlarged to 6000×, and the size of Si particles in the eutectic area was counted and averaged. The results are shown in Figs. S5 and S6. The shape factors for all investigated alloys are shown in Figs. 9–12. In the Al–Si binary alloy, with increasing Si content, the particle size of eutectic Si increases from 0.31 to 1.24 μm^2^, while no significant changes of the shape factor and phase spacing were observed. In contrast, in the Al–9Si–Sr alloy, with increasing Sr content, a significant change of the morphology of eutectic Si was observed. The particle size of eutectic Si decreases from 0.51 to 0.19 μm^2^, while the shape factor of eutectic Si increases from 0.610 to 0.705, as shown in Fig. 12. It should be noted that the above changes were observed when the Sr content was less than 56 ppm. With further increasing Sr content, the particle size and phase spacing of eutectic Si increase gradually, while the shape factor decreases slightly.

### Thermal and electrical conductivity of alloys

**Figure 13:**
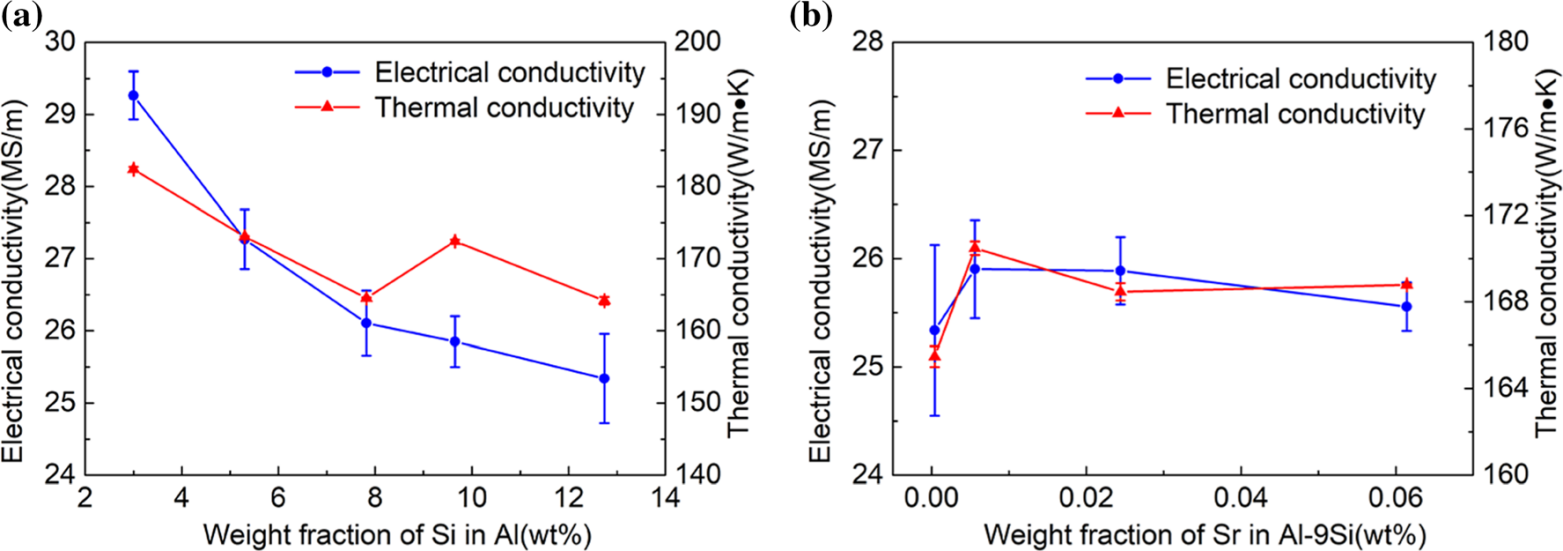
Electrical and thermal conductivity of **a** Al–Si binary alloys and **b** Al–9Si–Sr ternary alloys

Figure 13 shows the effects of Si and Sr contents on thermal conductivity and electrical conductivity. In the Al–Si binary alloy, with increasing Si content, the electrical conductivity decreases from 29.26 to 25.33 MSm^−1^, and the thermal conductivity decreases from 182.38 to 164.16 Wm^−1^ K. In Al–9Si–Sr ternary alloy, with increasing Sr content up to 56 ppm, the electrical conductivity increases from 25.34 to 25.90 MSm^−1^ and the thermal conductivity increases from 165.47 to 168.79 Wm^−1^ K. With further increasing the Sr content, the thermal conductivity and electrical conductivity decrease accordingly.

## Discussion

### Effects of Si and Sr elements on solidification microstructure

As shown in Figs. 4 and 6a, the α-Al grain size is dependent on the change of Si content. Furthermore, as listed in Table 2, the nucleation undercooling and recalescence increase with increasing Si content up to 9 wt%. As reported by Kearns et al. [15], the constitutional undercooling of various types of solutes in the Al solution can be superimposed on each other. Although high-purity Al was used in this paper, trace amounts of Fe (6 ppm), Cu (8 ppm), Mg (4 ppm) and Ti (< 1 ppm) are still present. Therefore, when the Si content is low, the Si in the melt does not react with these impurity elements to form intermetallic compounds. However, with increasing Si content, the compositional supercooling of the melt increases and promotes the refinement of the grains. The maximum supercooling degree Δ*T* can be expressed as (Eq. 3):

3 $$\Delta T = \Delta T_{{{\text{Si}}}} + \Delta T_{{{\text{im}}}} = \frac{{m_{{{\text{Si}}}} C_{{{\text{Si}}}} \left( {K_{{{\text{Si}}}} - 1} \right)}}{{K_{{{\text{Si}}}} }} + \sum \frac{{m_{{{\text{im}}}} C_{{{\text{im}}}} \left( {K_{{{\text{im}}}} - 1} \right)}}{{K_{{{\text{im}}}} }}$$

where Δ*T*_Si_ is the undercooling caused by Si, Δ*T*_im_ is the undercooling caused by other impurity elements, *m*_Si_ is the slope of the liquid-phase line of element Si, *C*_Si_ is the concentration of element Si, *K*_Si_ is the solute partition coefficient of element Si, *m*_im_ is the slope of the liquid phase line of other impurity elements, *C*_im_ is the concentration of other impurity elements and *K*_im_ is the solute partition coefficient of other impurity elements. In addition, with increasing Si content, the growth restriction factor also increases and inhibits the growth of α-Al grains. However, with further increasing Si content, the α-Al grain size increases, which can be attributed to the fact that Si with a high content in the melt reacts with other impurity elements and thereby forms intermetallic compounds, which decreases the effect of composition supercooling. It has been reported by Xu et al. that the presence of Si with a high content can react with Ti elements in the alloy to form Ti–Si, Ti–Al–Si and other intermetallic compounds either in the melt, which reduces the growth restriction factor caused by Ti, or on the surface of nucleation sites (i.e., TiB_2_ or TiC), which results in the inactivation of TiB_2_ or TiC particles for nucleation. The so-called “Si poisoning” phenomenon occurs [3], [3]. Moreover, with increasing Si content, the solidification onset temperature decreases and thus leads to an increase of superheat and intensifies the grain coarsening phenomenon at the constant pouring temperature, which is consistent with the variation of α-Al grain size with Si contents, as shown in Fig. 6.

In the Al–9Si–Sr ternary alloy, Sr and B can form SrB_6_ phase, which further reduces the heterogeneous nucleation efficiency of the impurity B element [17][17]. It should be noted that there is no significant effect of Sr on the grain size and the percentage of α-Al. Instead, a significant effect of Sr element on the modification of eutectic Si was observed. As shown in Fig. 10 and Fig. 12b, an appropriate amount (about 56 ppm) of Sr elements can effectively modify eutectic Si, but an excessive introduction of Sr elements (i.e., 614 ppm) has a negative effect on the modification of eutectic Si due to the formation of Al_2_Si_2_Sr intermetallic phase. In addition, there is an interaction between Sr and P via the formation of Sr_3_P_2_ instead of AlP [11], which decreases the nucleation temperature from 575.01 °C with 4 ppm Sr to 574.82 °C with 614 ppm Sr, as shown in Fig. 3 and listed in Table 3.

### Effects of Si and Sr elements on thermal conductivity of Al–9Si-based alloys

Free electrons are the main carriers for the electrical and thermal conduction of metals. When free electrons encounter vibrating atoms in motion, they are scattered by the vibrating atoms. The free electrons are affected by defects and phonon scattering to produce resistance and thermal resistance. Therefore, the main factor determining their electrical and thermal conductivity is therefore the mean free range of the free electrons. Since both thermal conductivity and electrical conductivity depend mainly on free electrons, there is a relationship between thermal and electrical conductivity, which satisfies the Wiedemann–Franz law (Eq. 4) [19], [19]:

4 $$\lambda = LT\sigma + c$$

where *L* is the Lorentz constant (2.1 × 10^−8^ W Ω K^−2^), *σ* is the electrical conductivity, *T* is the temperature and *c* is a constant (12.6 Wm^−1^ K). According to this equation, the thermal conductivity of the sample can be estimated empirically by electrical conductivity. Therefore, the mechanism of heat transfer can be simply discussed from the electrical conductivity theory.

In Al–Si binary alloys and Al–9Si–Sr ternary alloys, the eutectic Si, which is the main intermetallic phase, becomes the scattering center of the thermal energy carrier. Therefore, the morphology, size and distribution of eutectic Si have a great influence on the thermal conductivity of Al–Si alloys [5], [5], [5]. Since Al–Si binary alloys and Al–9Si–Sr ternary alloys have a relatively simple composition, these two alloys can be regarded as a composite of the primary α-Al phase and the eutectic Si phase and their thermal conductivity can be predicted. The thermal conductivity of alloys at room temperature can be predicted using mathematical models [19], [22]. Two classical models: the Maxwell model and the Hashin–Shtrikman (H–S) model, were used in this work. The Maxwell model derives the thermal conductivity of composite materials formed by random distribution of spherical particles in a continuous and uniform medium, which can be expressed as Eq. 5:

5 $$\lambda = \frac{{\lambda_{{\text{m}}} \left[ {2\left( {\lambda_{{\text{d}}} /\lambda_{{\text{m}}} - 1} \right)V_{{\text{d}}} + \frac{{\lambda_{{\text{d}}} }}{{\lambda_{{\text{m}}} }} + 2} \right]}}{{\left( {1 - \frac{{\lambda_{{\text{d}}} }}{{\lambda_{{\text{m}}} }}} \right)V_{{\text{d}}} + \frac{{\lambda_{{\text{d}}} }}{{\lambda_{{\text{m}}} }} + 2}}.$$

The H–S model can also be used to predict the thermal conductivity of isotropic heterogeneous material using both upper and lower bounds. The upper bound equates to the Maxwell model, and the lower bound is defined by Eq. 6:

6 $$\lambda = \frac{{\lambda_{{\text{d}}} \left[ {2\left( {\frac{{\lambda_{{\text{m}}} }}{{\lambda_{{\text{d}}} }} - 1} \right)V_{{\text{m}}} + \frac{{\lambda_{{\text{m}}} }}{{\lambda_{{\text{d}}} }} + 2} \right]}}{{\left( {1 - \frac{{\lambda_{{\text{m}}} }}{{\lambda_{{\text{d}}} }}} \right)V_{{\text{m}}} + \frac{{\lambda_{{\text{m}}} }}{{\lambda_{{\text{d}}} }} + 2}}.$$

In both Eq. 5 and Eq. 6, *λ* is the thermal conductivity, *V* is the volume fraction and the subscripts *m* and *d* are the matrix and dispersed phases, respectively. Here, the volume fraction of Al matrix (*V*_m_) and eutectic Si *(V*_d_*)* can be calculated according to their theoretical density and weight fraction in alloys using Thermo-Cal software. The thermal conductivity of the Si phase (25 Wm^−1^ K (λ_d_)) and pure Al (213.5 Wm^−1^ K (λ_m_)) has been reported [7]. Considering the determined contents of Si elements within α-Al matrix, the λ_m_ was also corrected accordingly in this work. It should be noted here that the determined contents of Sr elements within α-Al matrix are not taken into consideration because of its low level (Fig. 7b). Combined with the measured contents of Si within α-Al matrix (Fig. 7), the decrease in electrical conductivity of α-Al in all alloys can be calculated, and then, the corrected thermal conductivity of α-Al can be obtained based on Eq. 4.

**Figure 14:**
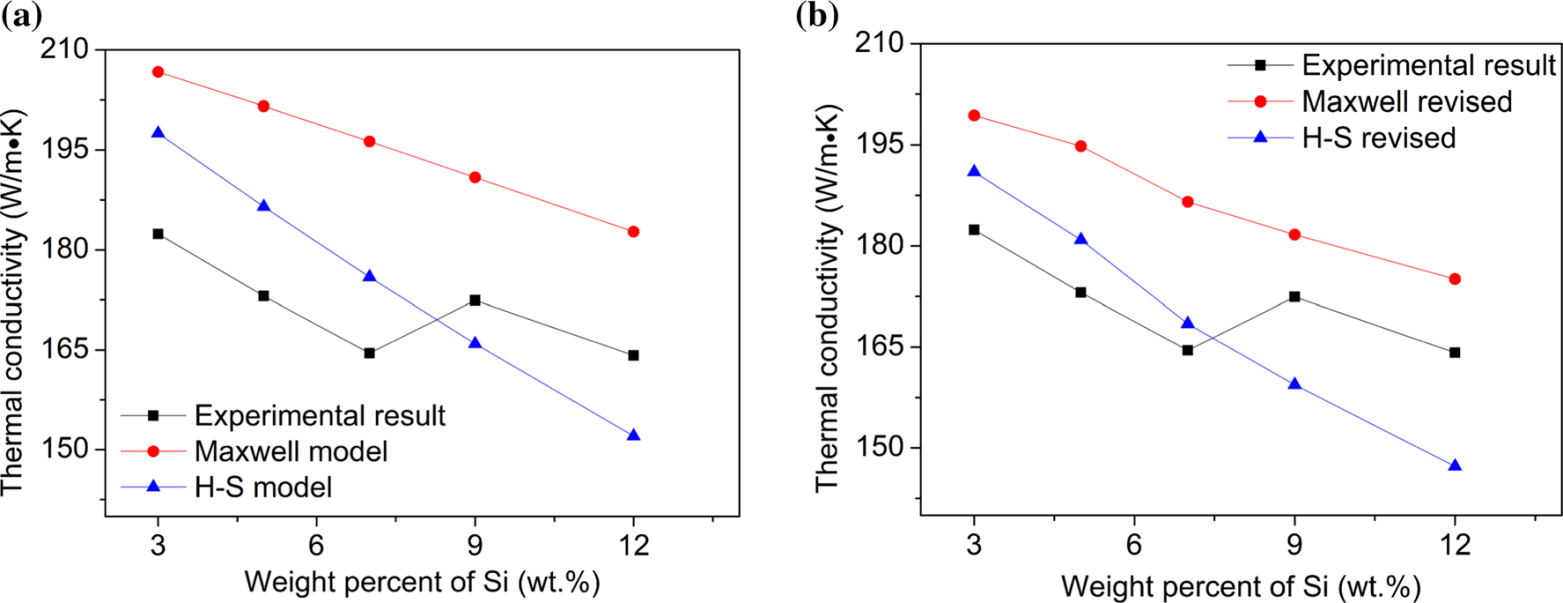
Comparison of the experimental and calculation data from **a** original model (Maxwell, H–S model) and **b** revised model of Al–Si binary alloys

**Figure 15:**
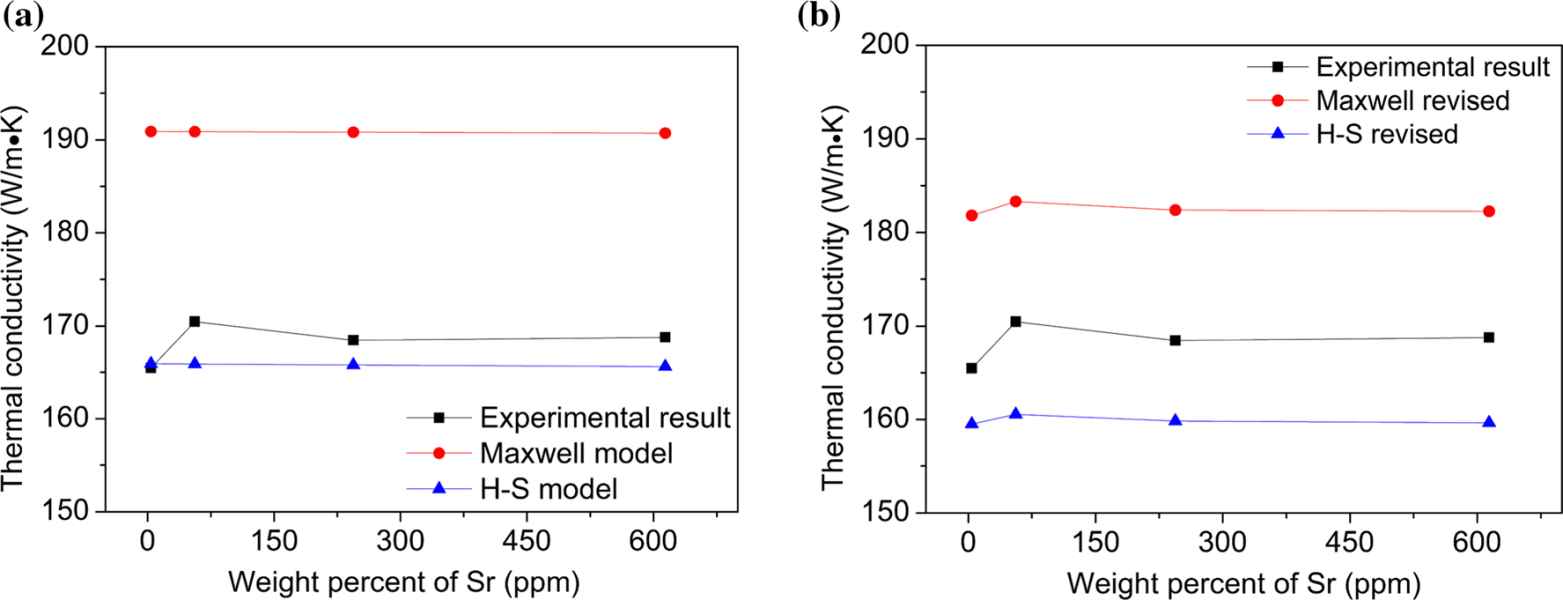
Comparison of the experimental and calculation data from **a** original model (Maxwell, H–S model) and **b** revised model of Al–9Si–Sr ternary alloys

The comparison of the experimental results with original model (Maxwell, H–S model) and revised model of Al–Si binary alloys and Al–9Si–Sr ternary alloys is shown in Figs. 14 and 15, respectively. In Al–Si binary alloys, the correlation between the experimental and the fitting result was 0.77 and 0.79 for both original Maxwell and H–S model, while for the revised models, the correlation was increased to 0.80 and 0.80, respectively. In Al–9Si–Sr ternary alloys, the correlation between the experimental and the fitting result was − 0.26 and − 0.26 for both original Maxwell and H–S model, while for the revised models, the correlation was increased to 0.91 and 0.83, respectively. The closer the correlation value is to 1, the more reliable the fitting result is. Clearly, it is of great necessity to correct the thermal conductivity according to the solid solution of the Al matrix. Furthermore, in Al–Si binary alloys, the average deviation between the experimental data and the H–S model was only 1.92 Wm^−1^ K, while that of Maxwell mode was 16.20 Wm^−1^ K. In Al–9Si–Sr ternary alloys, the average deviation between the experimental data and the H–S model was 8.43 Wm^−1^ K, while that of Maxwell mode was 14.14 Wm^−1^ K. Clearly, compared with the Maxwell model, the H–S model fits better with the measured values.

The difference between the measured values and the theoretical values can be interpreted by the fact that although high-purity Al and high-purity Si were used as raw materials in this paper, the alloy still contains a certain amount of impurity elements (especially for Ti), which leads to an additional decrease in the thermal conductivity of the alloy. Moreover, the theoretical calculation model only considers the role of the volume fraction of the intermetallic phase in the alloy and does not take the variation of the α-Al grain size into account. About the electron scattering, mismatch of the interfaces between Si lattice and the Al lattice might facilitate the electron scattering. In binary Al–Si alloys, at a low Si element content, the α-Al grain size decreases with increasing Si content. The increase of grain boundaries hinders the movement of free electrons and thereby reduces the thermal conductivity. With increasing Si content, the influence of Si element on thermal conductivity increases gradually, and the theoretical calculated value and the measured value are gradually close to each other. The thermal conductivity increases at about 7 wt% Si, which may be due to the increase of α-Al grain size and the decrease of grain boundaries caused by so-called Si poisoning. It can be seen from the EPMA results (Fig. 7a) that the solid solution degree of Si element in the α-Al matrix decreases when Si is less than 5 wt%, and therefore, the lattice distortion decreases, but the thermal conductivity decreases. With further increasing Si, the solid solution degree of Si element in the α-Al matrix increases, and therefore, the lattice distortion increases and the thermal conductivity decreases. This indicates that, compared with the solid solution degree of Si element in the α-Al matrix, the size and shape of eutectic Si particle are the dominant factors affecting the thermal conductivity. In Al–9Si–Sr ternary alloys, the addition of trace amounts of Sr element in the Al–9Si–Sr ternary alloy improves the thermal and electrical conductivity of the α-Al matrix. As described above, the introduction of Sr elements does not have a significant effect on the α-Al grain size, and the determined contents of Sr elements in the α-Al matrix remain essentially stable. Due to the same Si content (9 wt%) in the samples and the significant modification of eutectic Si caused by Sr elements, the particle size of eutectic Si significantly decreases and the shape factor significantly increases. The improvement of thermal and electrical conductivity can be therefore attributed to the change of the size and shape of eutectic Si particle.

**Figure 16:**
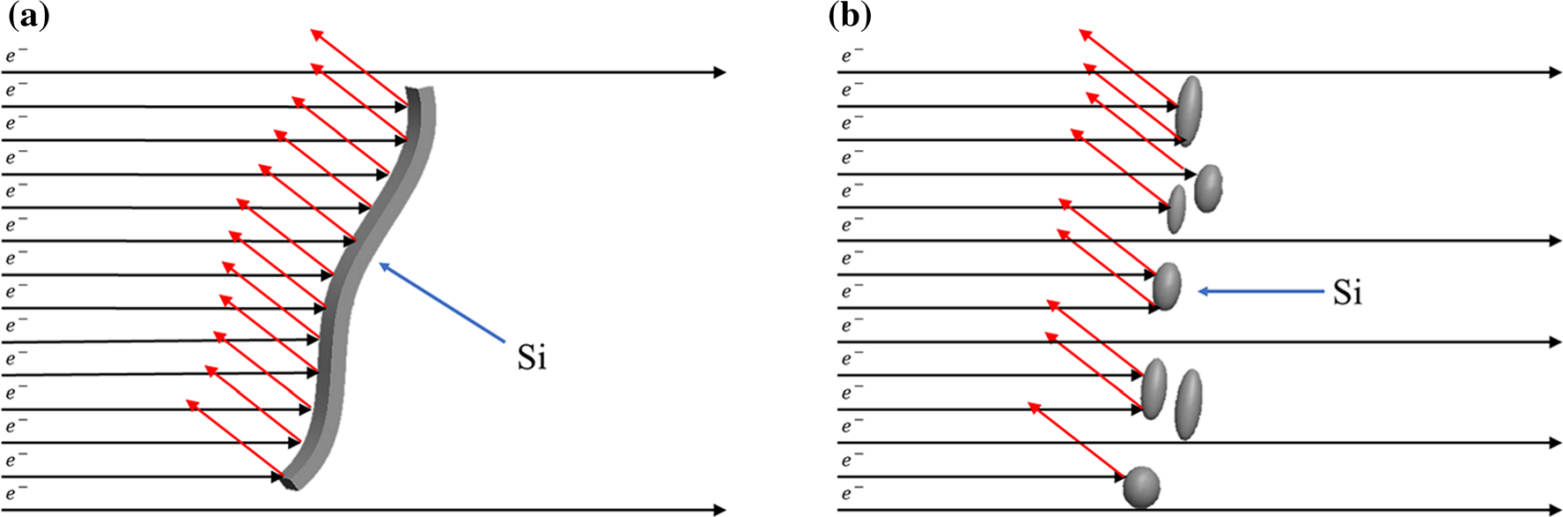
Schematic diagram of collision of free electrons with eutectic Si particles in **a** Al–Si binary alloys and **b** Al–9Si–Sr ternary alloys

Figure 16 shows the schematic diagram of the phenomenon of free electrons in collision with eutectic Si particles. The large plate-shaped Si particles clearly impede the movement of free electrons, while the modified Si particles have a reduced projection area on the α-Al substrate and thereby become to be less likely to impede the movement of free electrons. Therefore, the thermal and electrical conductivity of the modified Al–9Si–Sr ternary alloy increases. However, with further increasing Sr content, the morphological size and shape of eutectic Si particles deteriorate, which decreases the thermal and electrical conductivity.

## Conclusion

The effects of Si and Sr on solidification microstructure and thermal conductivity of Al–Si-based alloys were investigated. The following conclusions can be drawn:

1. The introduction of small amount of Si can promote the grain refinement of α-Al, but with further increasing Si content, the α-Al grain size increases and so-called Si poisoning phenomenon occurs. The increase of eutectic Si phase is responsible for the decrease in thermal conductivity and electrical conductivity of Al–Si binary alloys.
2. The introduction of Sr effectively modifies eutectic Si, reduces the projection area of eutectic Si particles on the α-Al substrate, and improves the thermal and electrical conductivity, but the introduction of excessive Sr elements leads to the deterioration of morphology and size of Si particles and decreases the thermal and electrical conductivity.
3. Two theoretical calculation models (the Maxwell model and the H–S model) were used to elucidate the effect of Si content on thermal conductivity. Compared with the Maxwell model, the H–S model fits better with the measured values.

## Supplementary Information

Below is the link to the electronic supplementary material.

Supplementary file1 (DOCX 11834 KB)

## Data Availability

The raw/processed data required to reproduce these findings cannot be shared at this time as the data also form part of an ongoing study.
