# Supplementary material for: Effects of Si and Sr elements on solidification microstructure and thermal conductivity of Al–Si-based alloys
Source: J Mater Sci. 2022 Mar 8;57(11):6428–44. doi: 10.1007/s10853-022-07045-7 (PMC8933373; doi:10.1007/s10853-022-07045-7)
Supplement: Supplementary file 1 — Supplementary file1 (DOCX 11834 KB) [file 10853_2022_7045_MOESM1_ESM.docx]

**Supplementary information**





**Fig. S1** SEM image of Al-Si binary alloys. (a) Al-3Si, (b) Al-5Si, (c) Al-7Si, (d) Al-9Si, (e) Al-12Si.


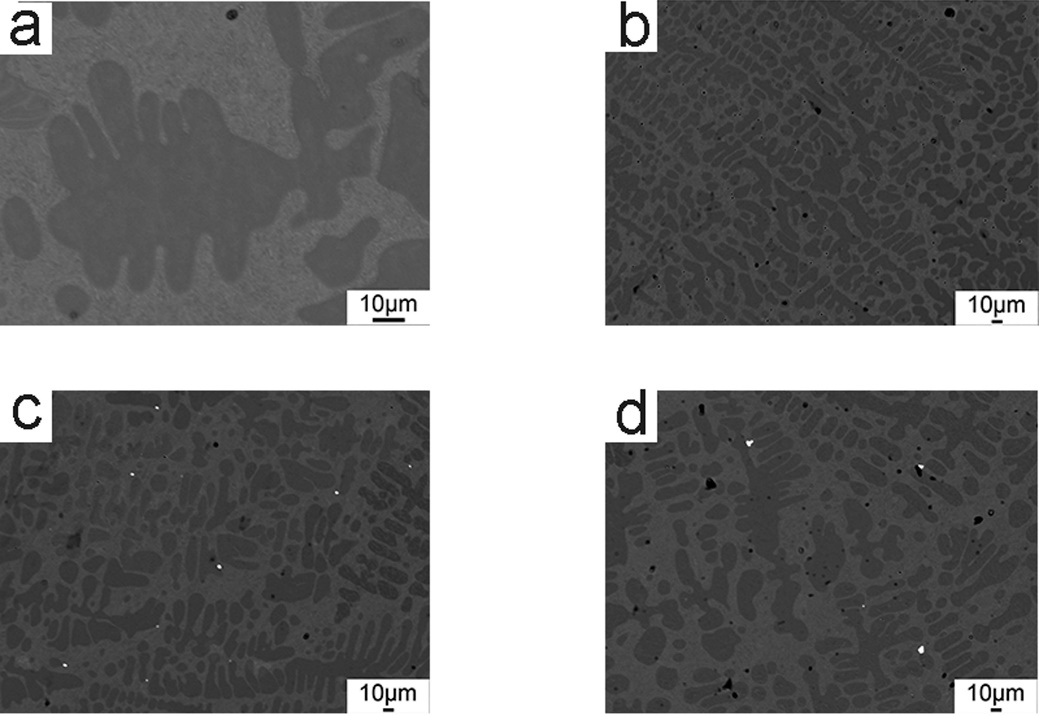


Fig. S2 SEM image of Al-9Si-Sr alloys with the addition of (a) 4ppm Sr, (b) 56ppm Sr, (c) 244ppm Sr, (d) 614ppm Sr.


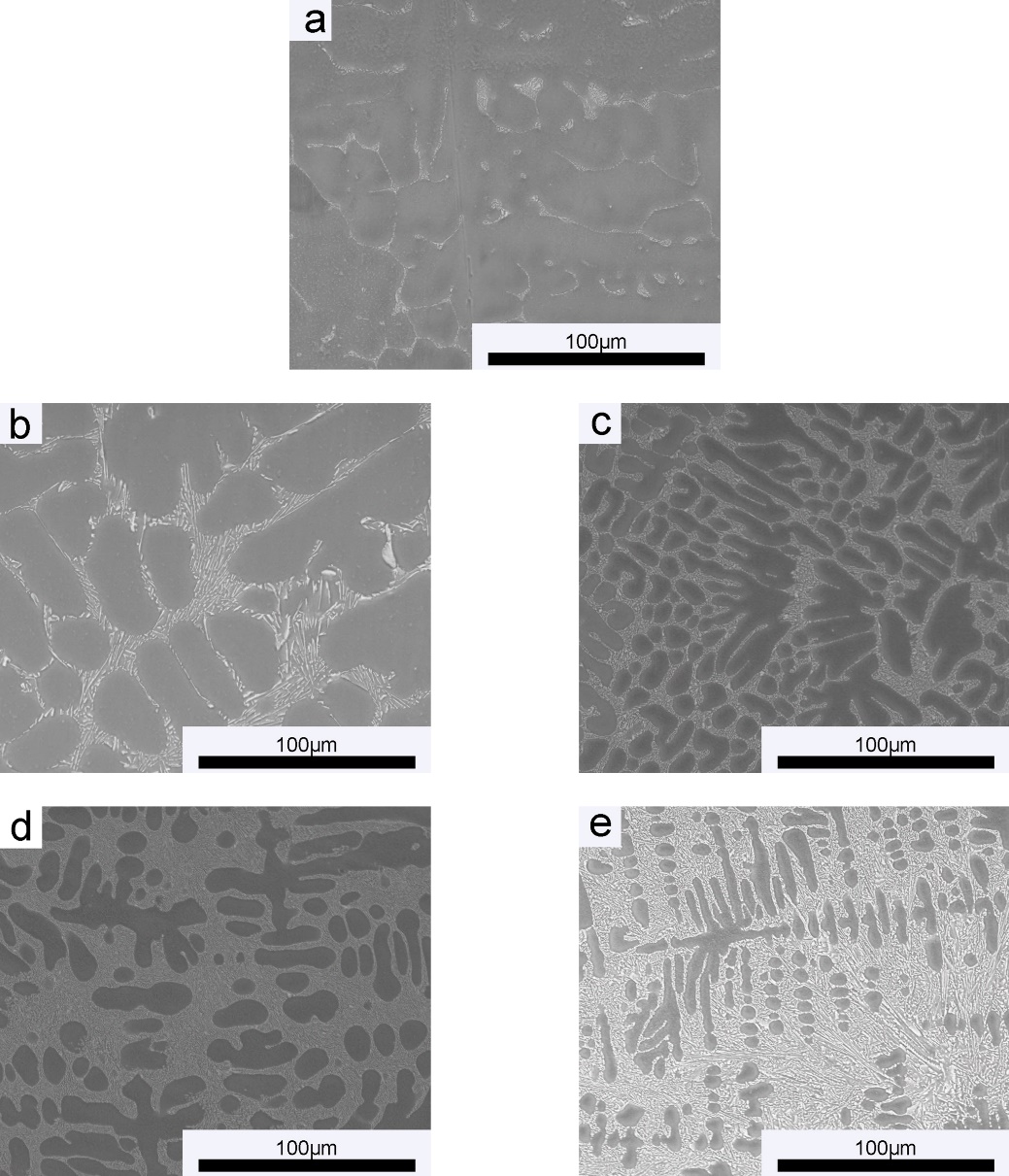


Fig. S3 SEM image of Al-Si binary alloy at 1000× magnification. (a) Al-3Si, (b) Al-5Si, (c) Al-7Si, (d) Al-9Si, (e) Al-12Si.


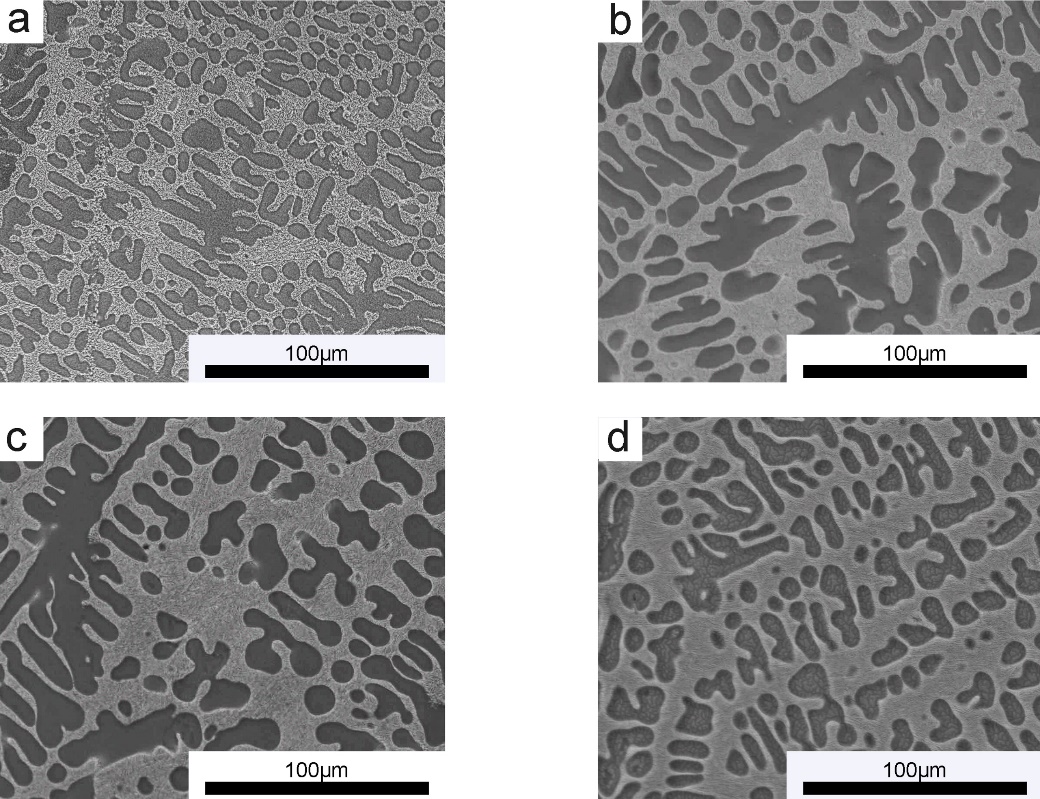


Fig. S4 SEM image of Al-9Si-Sr ternary alloy at 1000× magnification with the addition of (a) 4ppm Sr, (b) 56ppm Sr, (c) 244ppm Sr, (d) 614ppm Sr.


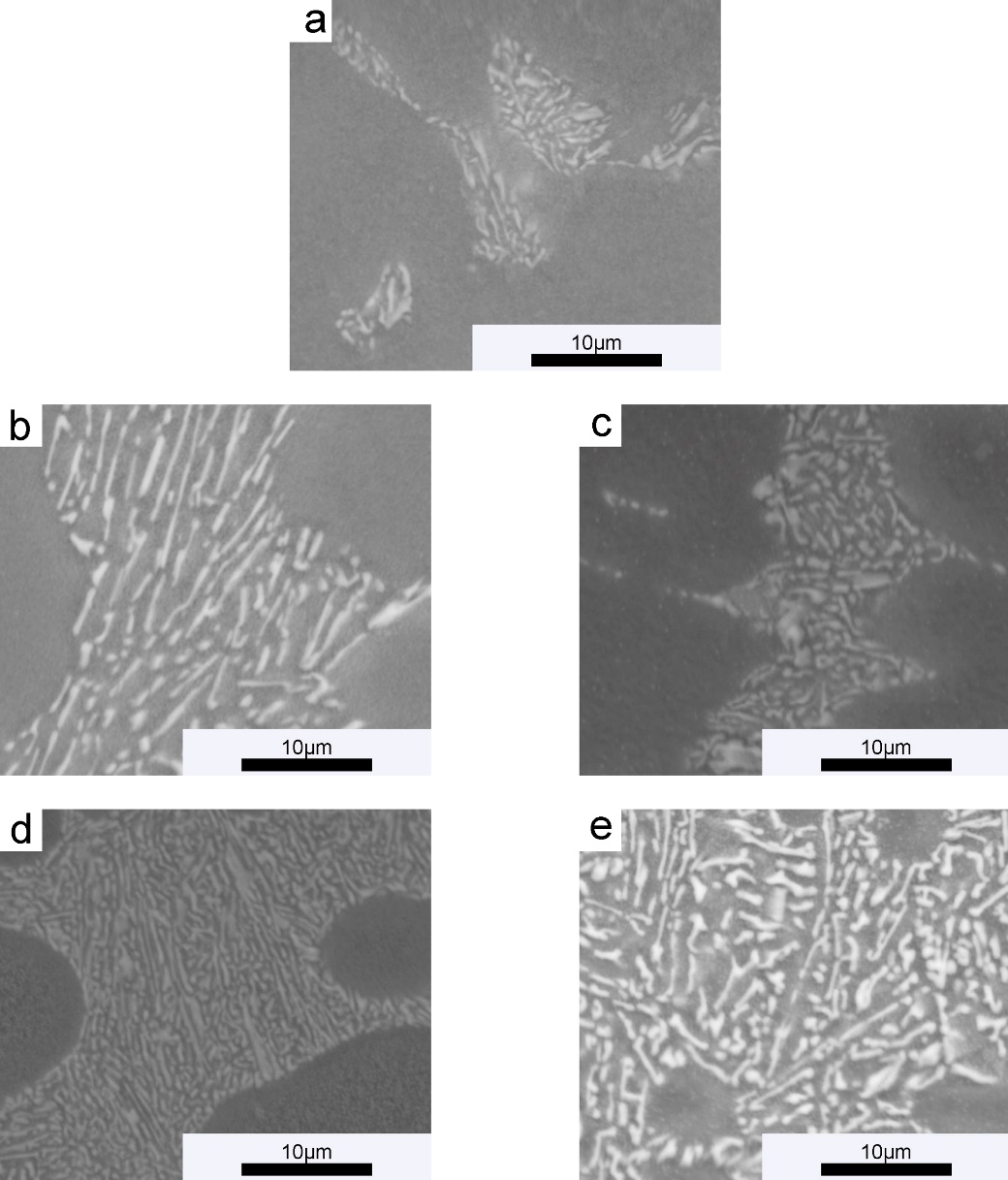


Fig. S5 SEM image of Al-Si binary alloys at 6000× magnification. (a) Al-3Si, (b) Al-5Si, (c) Al-7Si, (d) Al-9Si, (e) Al-12Si.


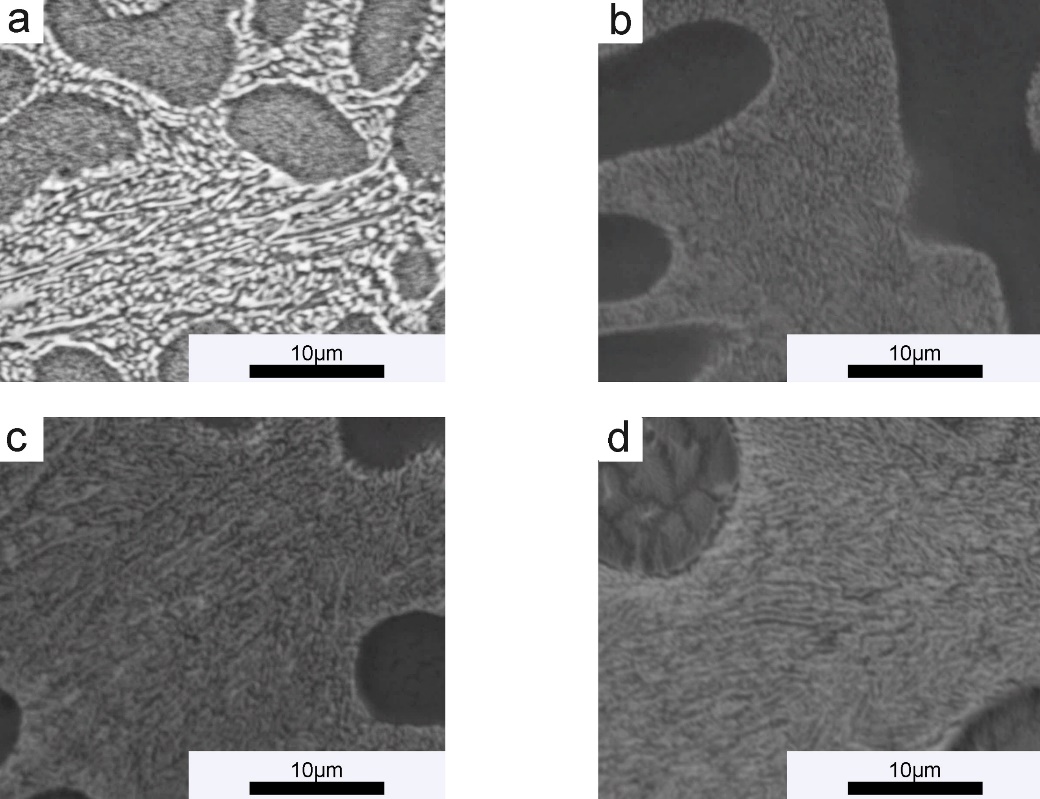


Fig. S6 SEM images of Al-9Si-Sr ternary alloy at 6000× magnification with the addition of (a) 4ppm Sr, (b) 56ppm Sr, (c) 244ppm Sr, (d) 614ppm Sr.
